# Supplementary material for: Stress-Driven Whisker Formation in Lithium Metal Batteries
Source: Nano Lett. 2025 Jul 14;25(29):11244–50. doi: 10.1021/acs.nanolett.5c01910 (PMC12291588; doi:10.1021/acs.nanolett.5c01910)
Supplement: Supplementary file 1 [file nl5c01910_si_001.pdf]

Supporting Information - Stress-driven whisker formation in lithium metal batteries

Martin Werres[1,2], Dariusz Niedziela[3], Arnulf Latz[1,2,4], and Birger Horstmann*[1,2,4]

Institute of Engineering Thermodynamics, German Aerospace Center (DLR), Wilhelm-Runge-Str. 10, 89081 Ulm, Germany

Helmholtz Institute Ulm (HIU), Helmholtzstr. 11, 89081 Ulm, Germany

Department of Flow and Material Simulation, Fraunhofer Institute for Industrial Mathematics, Fraunhofer-Platz 1, D-67663 Kaiserslautern, Germany

Department of Electrochemistry, University of Ulm, Albert-Einstein-Allee 47, 89081 Ulm, Germany

1. Model Parameters

Model parameters & physical constants	Value	Description
$D_{e^-}c_{e^-}$	1.5×10^{-17} mol/m^3	Diffusivity of electrons in SEI times concentration of electrons in the SEI near the Li interface
V_m^{Li}	13.02×10^{-6} m^3/mol	Molar volume of Li
V_m^{SEI}	95.86×10^{-6} m^3/mol	Molar volume of the SEI
F	96485 C/mol	Faraday constant
C	1.59×10^{6} Pa s	Shear-thinning dimensionality constant
n	0.1515	Stress exponent
σ_{yield}	1×10^{7} Pa	Yield stress
m	100000	Herschel-Bulkley regularization parameter
ρ	534 kg/m^3	Lithium density
$\rho_{Electrolyte}$	1241 kg/m^3	Electrolyte density
$\eta_{electrolyte}$	3.045×10^{-3} Pa s	Electrolyte viscosity

SI Table 1: Table of model parameters and constants.

The model parameters C and n are derived from the experimental observations of LePage et al. [1]. They observed that the secondary creep phase can be described by the power-law:

$$\frac{\dot{\epsilon}}{\mathrm{s}^{-1}} = A_c \left(\frac{\sigma}{\mathrm{Pa}}\right)^m \exp\left(-\frac{Q_c}{RT}\right),$$

where $\dot{\epsilon}$ is the strain rate, σ is the uniaxial stress, and m, A_c, and Q_c are material parameters determined by their experiments. They determined the coefficients to be $m = 6.6$, $A_c = 7.1 \times 10^{-37}$, and $Q_c = 37\ \mathrm{kJ\ mol^{-1}}$.

With this parameters, we can calculate an effective extensional viscosity $\mu_{\text{elongational}} = \sigma/\dot{\epsilon}$:

$$\frac{\mu_{\text{elongational}}}{\mathrm{Pa\cdot s}} = \frac{\sigma}{\dot{\epsilon}} = A_c^{-\frac{1}{m}} \exp\left(\frac{Q_c}{mRT}\right)\left(\frac{\dot{\epsilon}}{s^{-1}}\right)^{\frac{1}{m}-1} .$$

At room temperature (T=293.15K), the elongational viscosity is:

$$\frac{\mu_{\text{elongational}}(T = 293.15K)}{\text{Pa} \cdot \text{s}} = 2.99 \times 10^6 \left(\frac{\dot{\epsilon}}{s^{-1}}\right)^{\frac{1}{m}-1} .$$

We make the assumption that the extensional viscosity can be related to the shear viscosity via the Trouton ratio:

$$Tr = \frac{\mu_{\text{elongational}}}{\mu_{\text{shear}}} \approx 3 .$$

The shear rate is the second invariant of the strain-rate tensor:

$$\dot{\gamma} = \sqrt{2\epsilon : \epsilon} .$$

For $\epsilon = \begin{pmatrix} -0.5 & 0 & 0 \\ 0 & -0.5 & 0 \\ 0 & 0 & 1 \end{pmatrix} \dot{\epsilon}$, $\dot{\gamma} = \sqrt{3}\dot{\epsilon}$. We use this to express the shear viscosity as

$$\frac{\mu_{shear}}{\text{Pa} \cdot \text{s}} = \frac{1}{3} \cdot 3^{\frac{1-\frac{1}{m}}{2}} \cdot 2.99 \times 10^6 \left(\frac{\dot{\gamma}}{s^{-1}}\right)^{\frac{1}{m}-1} = 1.59 \times 10^6 \left(\frac{\dot{\gamma}}{s^{-1}}\right)^{\frac{1}{m}-1} .$$

Thus, the model parameters are $C = 1.59 \times 10^6$ Pa $\cdot$ s and $n = \frac{1}{m} = 0.1515$.

2. Detailed Setup

The simulation domain is setup with

$$\Delta x = \Delta y = \Delta z = 10 \text{ nm}$$

and a cross-section of the simulation domain is shown in Figure SI 1. The white space is void and not solved for. The parts of the simulation domain which don’t have a connection to a different domain are bounded by no-slip wall conditions.

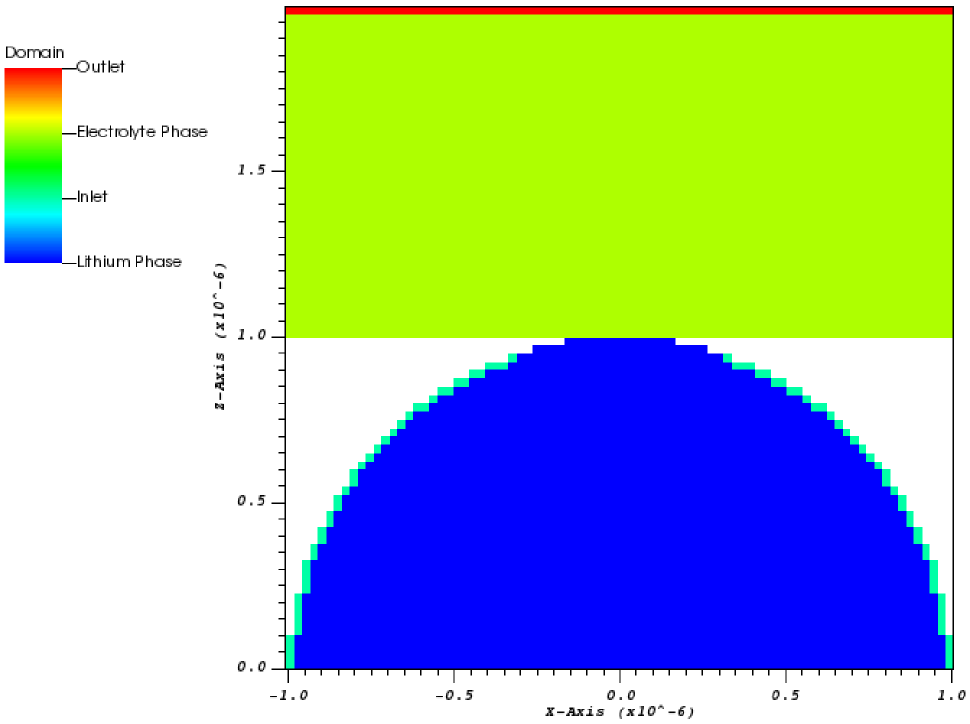

SI 2: Cross-section of the simulation domain setup. The initial Herschel-Bulkley phase is blue, the inlet is turquoise, the electrolyte phase is green, and the outlet is red.

3. Impact of yield stress on pressure distribution

Since we can only estimate the yield stress of Lithium on this small length scales, we studied how the predicted pressure distribution changes for a different yield stress. The bulk yield stress of lithium is reported to be between 0.57 and 1.26 MPa, we chose 1 MPa as a comparison. The resulting pressure distribution is depicted in Figure SI 2. Comparing this to the results with σ_{yield} =10 MPa, the stress distribution looks similar but the predicted pressure values are overall smaller by a factor of approximately 3.

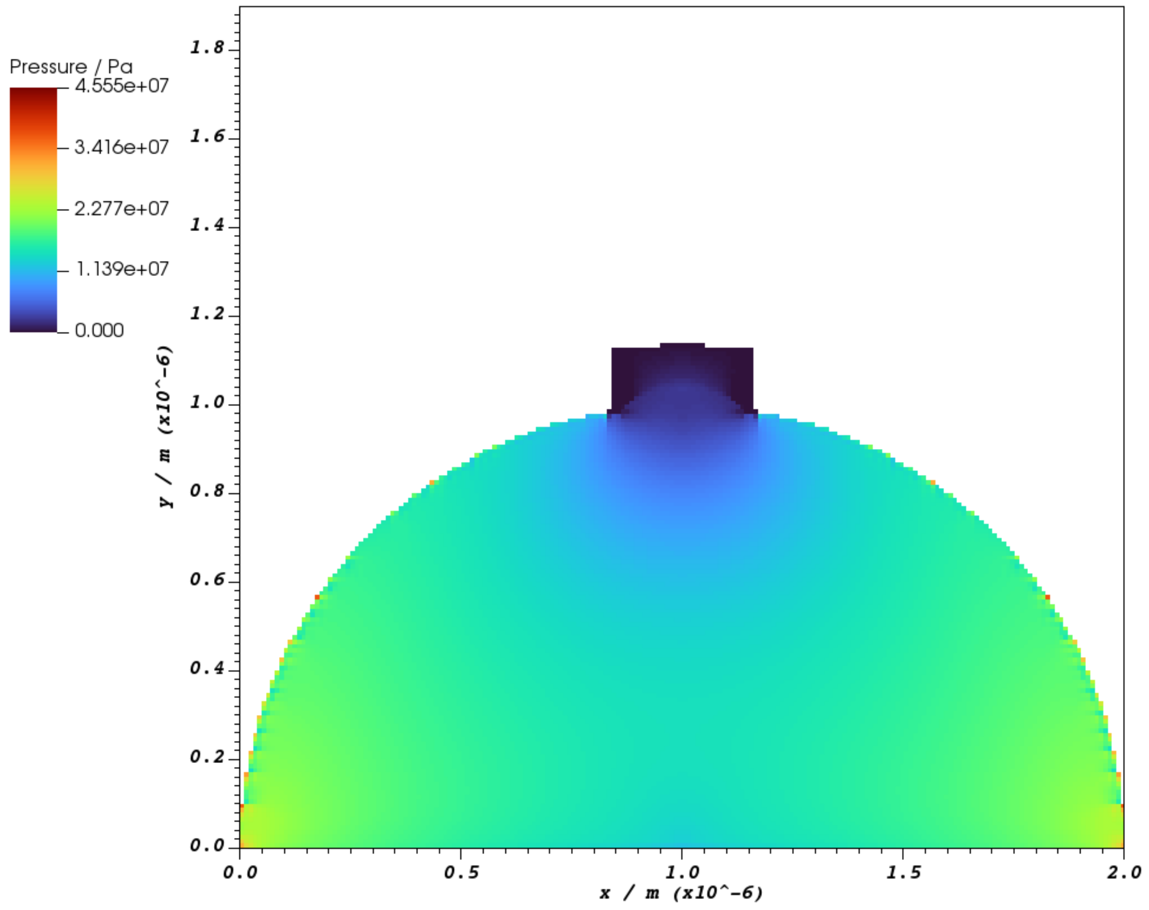

SI 3: Predicted pressure distribution with yield stress = 1 MPa, after t = 0.15s.

References

[1] LePage, W. S. *et al.* Lithium Mechanics: Roles of Strain Rate and Temperature and Implications for Lithium Metal Batteries. *J. Electrochem. Soc.* **166**, A89–A97 (2019)
